# Social and economic value of Portuguese community pharmacies in health care

**DOI:** 10.1186/s12913-017-2525-4

**Published:** 2017-08-29

**Authors:** Jorge Félix, Diana Ferreira, Marta Afonso-Silva, Marta Vargas Gomes, César Ferreira, Björn Vandewalle, Sara Marques, Melina Mota, Suzete Costa, Maria Cary, Inês Teixeira, Ema Paulino, Bruno Macedo, Carlos Maurício Barbosa

**Affiliations:** 1 Exigo Consultores, Lisbon, Portugal; 2 Centre for Health Evaluation & Research, National Association of Pharmacies (CEFAR), Lisbon, Portugal; 3 Portuguese Pharmaceutical Society, Lisbon, Portugal; 4 Department of Drug Sciences, Faculty of Pharmacy, University of Porto, Porto, Portugal

**Keywords:** Community pharmacies, Pharmacist, Health care, Social value, Economic value

## Abstract

**Background:**

Community pharmacies are major contributors to health care systems across the world. Several studies have been conducted to evaluate community pharmacies services in health care. The purpose of this study was to estimate the social and economic benefits of current and potential future community pharmacies services provided by pharmacists in health care in Portugal.

**Methods:**

The social and economic value of community pharmacies services was estimated through a decision-model. Model inputs included effectiveness data, quality of life (QoL) and health resource consumption, obtained though literature review and adapted to Portuguese reality by an expert panel. The estimated economic value was the result of non-remunerated pharmaceutical services plus health resource consumption potentially avoided. Social and economic value of community pharmacies services derives from the comparison of two scenarios: “with service” versus “without service”.

**Results:**

It is estimated that current community pharmacies services in Portugal provide a gain in QoL of 8.3% and an economic value of 879.6 million euros (M€), including 342.1 M€ in non-remunerated pharmaceutical services and 448.1 M€ in avoided expense with health resource consumption. Potential future community pharmacies services may provide an additional increase of 6.9% in QoL and be associated with an economic value of 144.8 M€: 120.3 M€ in non-remunerated services and 24.5 M€ in potential savings with health resource consumption.

**Conclusions:**

Community pharmacies services provide considerable benefit in QoL and economic value. An increase range of services including a greater integration in primary and secondary care, among other transversal services, may add further social and economic value to the society.

**Electronic supplementary material:**

The online version of this article (doi:10.1186/s12913-017-2525-4) contains supplementary material, which is available to authorized users.

## Background

Health care systems have suffered several challenges due to the financial crisis that affected Europe in recent years [1]. Portugal, amongst other countries, has implemented several measures in order to curb health care costs [2]. As a consequence, from 2011 to 2014, total health expenditure decreased more than 6%, to a level of 15,681.9 million euros (M€) in 2014, which represented, approximately, 9.1% of the Gross Domestic Product (GDP) [3]. The substantial reduction of Public Health expenditure may have a direct impact on health care services, originating consumption restraints and ultimately limiting access to health care [4–7].

Community pharmacists are major contributors to health care system across the world. The role of the pharmacist in the community has evolved throughout the years, shifting from medicines to a patient-centred approach with increased provision of clinical pharmacy services [8–10]. Pharmacists are particularly qualified to provide pharmacotherapeutic counselling, monitor therapy outcomes, contribute to decrease the risk for unintentional adverse events and prevent drug interactions [11–15]. In addition, community pharmacists are uniquely positioned within the health care system, having established a widespread network of services that provide direct outpatient care.

Hence, it is vital to promote health interventions that contribute, not only to a widespread access to health care, but also to more efficient resource allocation.

Several systematic literature reviews and meta-analysis that evaluate the impact of community pharmacies services in health care have been conducted, revealing positive impacts on patient’s outcomes, as well as economic benefits [10, 16–18]. Specifically, services provided in cardiovascular diseases showed a significant impact on systolic and diastolic blood pressure, with reductions of 6.1 mmHg and 2.5 mmHg, respectively [19]. Moreover, there is evidence suggesting that reducing diastolic blood pressure (−2 mmHg) results in a 6% and 15% risk reduction of coronary heart disease and stroke and transient ischemic attacks, respectively [20]. Pharmaceutical services in diabetes, asthma, smoking cessation or syringe-exchange programs have also demonstrated health gains in the enrolled population [11, 21–23]. Notwithstanding the positive clinical benefits, these services also represent cost-effective measures, leading to lower direct medical costs [21, 24–26].

In Portugal, all community pharmacies are privately owned and, similarly to other countries, their core activity is to dispense prescription and non-prescription medicines. Even tough community pharmacies hold the exclusivity of the first, since 2005, dispensing of the latter was authorised outside of community pharmacies. Since 2007, the range of pharmaceutical services provided has been widened with the introduction, for example, of immunization services (i.e., vaccines not included in the Nacional Vaccination Plan), disease management campaigns, health campaigns and home care support. It should be noted that community pharmacies in Portugal are not reimbursed by the National Healthcare System for the provision of any of these services, but patients pay out-of-pocket for some of them [27, 28].

A study conducted by Félix et al. [29] evaluated the impact of community pharmacies’ participation in the national syringe-exchange program. This study estimated that 7000 new human immunodeficiency virus (HIV) infections were avoided during the first 8 years of the program, resulting in overall economic savings of 400 M€ in health care costs related to HIV [29]. Nonetheless, to date, no study has been conducted in Portugal to globally estimate the social and economic value of the full range of services delivered by community pharmacists. Therefore this study aimed to estimate the social and economic benefits of current and potential future community pharmacies services provided by pharmacists in health care, in Portugal.

## Methods

The conceptual model developed evaluated the impact of community pharmacies services through the comparison of two scenarios: “with service” and “without service”. The model was designed to consider micro outcomes to evaluate the effectiveness of each service and macro outcomes, such as quality-adjusted life years (QALYs) and health resource consumption, to allow the assessment of the aggregated social and economic value of all the services.

The definition of the list of services included required the identification of services already provided by the Portuguese community pharmacies (current services) and potential services that could be implemented (potential future services).

In order to parameterize the model, a literature review was conducted to identify the specific outcomes of every service evaluated, including disease specific, QoL and resource consumption.

The model was adapted and validated to the national reality by an expert panel.

**Fig. 1:**
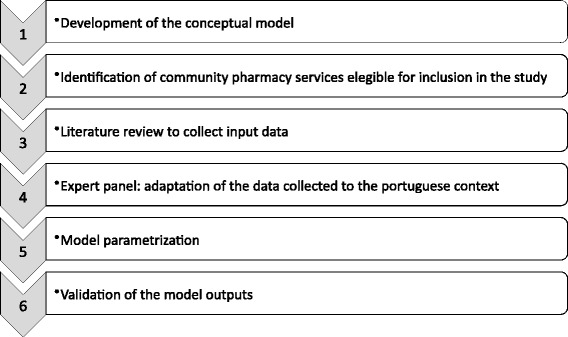
Summary study methodology

Fig. 1 depicts briefly the steps taken in this project.

### Conceptual model

**Fig. 2:**
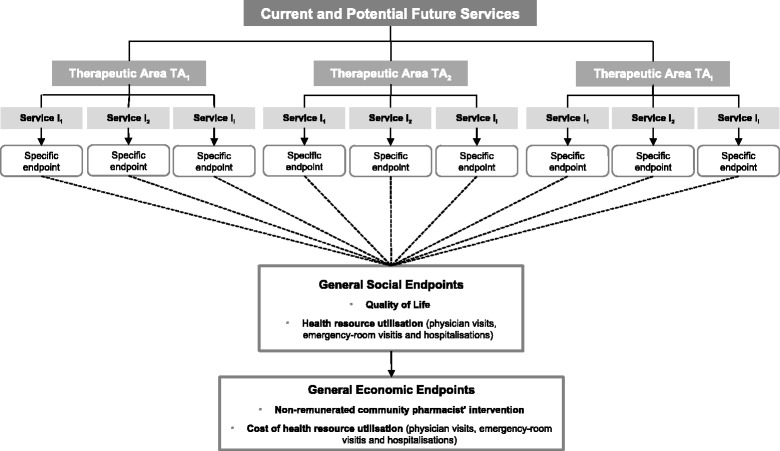
Conceptual Model

A decision model was developed to estimate the social and economic value of community pharmacies services (Fig. 2). The comparison of both scenarios involved several outcomes divided in three classes: therapeutic area-specific, social and economic. Therapeutic area-specific outcomes consisted, for example on blood pressure in hypertension or glycated haemoglobin in diabetes.

QoL and health care resource utilisation were regarded as social outcomes; and economic outcomes included non-remunerated community pharmacies services costs and health care resource utilisation costs avoided (Fig. 2).

Finally, the specific, social and economic evaluations were the result of the difference between the scenario with and without services delivered at community pharmacies. Services that are not entirely focused on one particular clinical outcome but instead have a broader range were evaluated separately and only included the economic value.

The model adopted a 1-year time horizon (2014) and the societal perspective.

### Pharmaceutical services included in the model

Current services are already being provided by Portuguese community pharmacies, for example, disease management programs, smoking cessation, specific counselling, among others (Additional file 1: Table S1). These were retrieved from data from Centre for Health Evaluation & Research (CEFAR) and from public documents that specify the services that can be performed in pharmacies in Portugal [27, 30, 31]. Potential future services comprised services that could be provided in Portuguese community pharmacies, including services that were once provided by Portuguese community pharmacists but have been discontinued, services performed in other countries and not yet implemented in Portugal, services tested as part of pilot studies that have also been discontinued and interesting services that should be developed by national community pharmacies, some already included in a recent legal order issued by the Portuguese government (Additional file 2: Table S2) [32].

Finally, the services which included a broader range of action included: medicines wastage, syringe exchange program, medicines disposal program (*Valormed*), research projects and pharmaceutical students’ curricular internships.

### Literature search and data extraction

An extensive comprehensive search in MEDLINE (PubMed) was conducted in order to identify the disease specific and economic outcomes of pharmaceutical services. The search included articles published before July 2015 studying pharmaceutical services in the community pharmacy context. The search strategy included terms for the pharmacist or community pharmacy, pharmaceutical services and social and economic endpoints (Additional file 3: Table S3). Language restrictions were adopted for English and Portuguese articles. A search in Google and GoogleSchoolar was also conducted to find any additional or grey literature. In addition, studies reporting on national community pharmacies services presented at congresses or available as reports were requested to CEFAR.

Inclusion criteria included services or observational studies conducted either in community pharmacies with results (clinical, social or economic) before and after the service implementation or in participants’ groups with and without service. Studies included in the quantitative synthesis had to report endpoints of interest (clinical, social and/or economic). Unoriginal work (reviews, meta-analysis, editorials and letters to the editor), pharmaceutical services performed in hospitals or in the health maintenance organization setting, or projects conducted by pharmacy students were excluded.

Whenever the abstract had insufficient detail to assess for inclusion criteria, the record was included for full-text revision. After reviewing titles and abstracts, a hard copy of each record that met inclusion criteria was obtained for full-text review.

Data from studies that met inclusion criteria were extracted from each record: author, year of publication, condition targeted, number of patients enrolled and outcomes reported (clinical, social or economic) before and after the service implementation or in the control and intervention groups.

### Expert panel

After data extraction, a synthesis of the reviewed data was presented to a national expert panel of seven pharmacists, specialists in pharmaceutical care, and two physicians. The pharmacists were selected based on their experience in pharmaceutical services in community pharmacies and are all considered experts in this field; additionally, the panel presented geographic heterogeneity. Physicians selected present deep knowledge and vast experience in the relation between community pharmacies and primary care, and as so were able to quantify the impact of pharmaceutical services in patients’ QoL and at an economic level. All the experts gave their oral consent for participating in this study.

Experts were asked to validate and adjust collected data to the Portuguese reality according to their experience and expectations, and whenever data was unavailable, experts were asked to suggest an estimated value based on their experience. Data was collected individually from each expert in order to reflect the possible heterogeneity in their practice; no consensus was aimed to be achieved.

A meeting was held with the pharmacists’ expert panel in order to discuss the pharmaceutical services included in the model, covered population, time spent by a pharmacist in a specific service, number of services performed and specific endpoints related to the therapeutic area, whenever this information was lacking in the literature or not available from CEFAR data. For this meeting, data collection was obtained with an electronic system, which required the experts to answer the questions in a device connected to the software. After all experts had provided their responses, aggregated mean values and standard deviations or frequency of responses, were automatically presented in the computer screen. Invited physicians were interviewed individually regarding data about health resource consumptions and QoL. Both meetings with the physicians were conducted using a structured questionnaire where their answers were noted. No interview guide was developed, but all the meetings were conducted by the same person in order to guarantee homogeneity in data collection.

Following the expert panel meeting, mean estimated values were incorporated in the model. Services considered irrelevant in the Portuguese context by the pharmacist experts were excluded from the model.

### Resources utilisation and costs

The annual number of community pharmacies services was directly obtained from the study conducted by Pita Barros et al. [33], which was further completed with data directly requested to the authors. The amount of annual services was then divided by the mean number of annual services per patient, thus obtaining the covered population. Whenever the amount of a specific service was not available in the study by Pita Barros et al. [33], data was provided by CEFAR. CEFAR possesses data from a representative sample of community pharmacies from which the annual number of pharmaceutical services and their respective cost for the patient can be extracted; however, not all community pharmacies register services provided, particularly when services are free of charge. If the amount of a specific service was not available in the study by Pita Barros et al. neither in CEFAR data, the estimated mean number of services provided per year was elicited by the expert panel of pharmacists.

The economic value provided by non-remunerated pharmaceutical services derived from the pharmacy cost per minute (0.53€) [34] multiplied by the time spent on each service, obtained from the study conducted by Pita Barros et al. [33], and by the estimated number of services performed. Whenever adequate, the resulting cost was then subtracted by the patients’ charged price, obtained from CEFAR data [34, 35].

Concerning the economic impact of pharmaceutical services, the cost of a physician visit was estimated considering the costs and the number of physician visits performed. The cost of each physician visit used (67.02€) is a weighted average [36] of the cost of primary care general practitioner visit in Portugal (68.60€) [37] and the cost of specialist visit (63.19 €) in secondary care (the cost of a secondary care visit was retrieved from a Ministry of Health source which stipulates the agreed cost to pay the hospitals for that visit, this value may be lower than the actual value of the visit) [38].

A similar cost estimating process was used for the emergency-room visits. Hospital funding regarding emergency-rooms is allocated based on three types of emergency services and respective funding levels [38]. The cost of each level was then multiplied by the number of emergency-rooms visits performed on the hospitals of each level [36]. As a result, the average weighted cost incorporated in the model for an emergency-room visit was 71.86 €.

The average cost of hospitalisation was directly collected from the Contract Program of Central Administration of the Health System (2,120.28 €), except for hospitalisation related to diabetes, asthma, hypertension, blood coagulation variations, chronic obstructive pulmonary disease (COPD) and dyslipidaemia [38]. For these situations, a specific cost was estimated for each therapeutic area, linking the International Classification of Diseases (ICD-9) with the respective most frequent codes of the diagnosis-related groups (DRG). The proportion of selected DRG and the associated price were validated by the physician experts.

The social and economic impact in specific chronic conditions was included in the value provided by overall current community pharmacies services.

Other current services (syringe-exchange program, medicines disposal program and research projects) were evaluated according to the time spent by the pharmacist multiplied by the pharmacy cost per minute [34]. The additional value for money of the syringe-exchange program was estimated in a previous study, conducted in Portugal in 2002 [29] and updated using the Consumer Price Index variation for 2014 (the last year available) and then multiplied by the number of syringes exchanged when community pharmacies were not involved in the syringe exchange national program (year 2013).

The economic value associated with the medicines wasted management took into account the role of pharmacists in the promotion of patient adherence and consequently the mean number of packages consumed due to the service. The latter was then multiplied by the cost per package attributable to non-adherence, previously described in the literature (2.85 €) [39].

The non-remunerated value related to the services of community pharmacies in pharmaceutical students’ curricular internships was assessed by allocating the proportion of faculty fees corresponding to the pharmaceutical students’ curricular internship in the pharmacy, which is currently granted to the faculties.

## Results

### Literature review

**Fig. 3:**
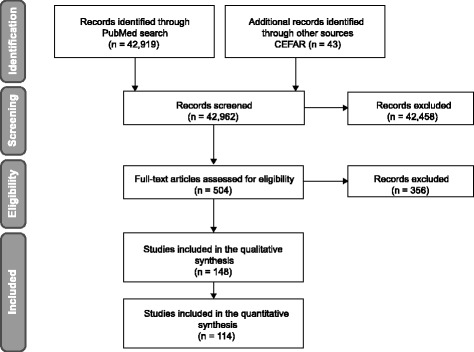
PRISMA flow-chart of literature review

The literature review identified 42,919 records in MEDLINE (PubMed) and 43 additional records (posters and reports) were provided by CEFAR (Fig. 3). The initial screening process, through title and abstracts reading, excluded mainly opinion articles or articles reporting secondary data (derived from literature reviews or meta-analysis). The most common reasons for exclusion after full-text reading were records of services provided by hospital pharmacies, health maintenance organizations, services conducted by pharmacy students or not evaluating services previously selected to be included in the model.

### Expert panel

The expert panel decided consensually that some proposed services should be removed from the model. In current services, inspection of first aids kits was removed, due to the low availability of this service in Portuguese community pharmacies. Also, in potential future services, alcohol cessation and collection of biological products were removed since they were considered a multidisciplinary service not performed exclusively by pharmacists.

### Current services

The social and economic value of community pharmacies services is depicted in Table 1. The added value of community pharmacies services was the result of the difference between a scenario with and a scenario without the evaluated services. Table 1 also identifies the estimated population covered in total (which represents 40.3% of the Portuguese population), the total number of services and total pharmaceutical time spent to provide such services.

**Table 1:** Social and economic value of the current community pharmacies services

*Current services*			
	With service	Without service	Difference
Population Covered (*n*)	4,180,190	4,180,190	0
Estimated number of pharmaceutical services (*n*)	120,675,438	0	120,675,438
Time spent in services (*hours*)	11,087,135	0	11,087,135
Social Value			
Quality of Life			
Quality of Life (growth rate, %)	0.813	0.751	0.062 (+8.3%)
Quality adjusted life years	3,399,191	3,138,946	260,245
Health Resource Utilisation (n)	20,818,175	26,853,745	−6,035,571
Physician visits (n)	17,881,598	23,872,706	−5,991,108
Emergency-room visits (n)	2,871,217	2,893,271	−22,054
Hospitalisations (n)	65,359	87,768	−22,409
Economic Value			
Total cost (€)	1, 192.2 M€	2, 071.9 M€	−879.6 M€
Community pharmacy (€)			
Community pharmacist’ service (monetary compensated and non-monetary compensated)	−352.1 M€	0.0 €	−352.1 M€
Patients	10.0 M€	0.0 €	10.0 M€
Cost of Health Resource Utilisation (€)			
Physician visits	1, 198.4 M€	1, 599.9 M€	−401.5 M€
Emergency-room visits	206.3 M€	207.9 M€	−1.6 M€
Hospitalisations	134.4 M€	179.4 M€	−45.0 M€
Other costs (€)			
Medicines waste management	14.9 M€	85.0 M€	−70.1 M€
Syringe exchange program	−6.3 M€	−0.3 M€	−6.0 M€
Others	−13.4 M€	0	−13.4 M€

M€, Million euros

It is estimated that current community pharmacies services represent a gain of 8.3% in QoL, providing 260,245 additional QALYs to the covered population in the year of 2014 (Table 1). The aggregated economic value provided by current services represents an added value to the society of 879.6 M€ (Table 1). This value is further divided in three main areas: 50.9% related to health care resource utilisation (448.1 M€), 38.9% from non-remunerated pharmaceutical services related with specific therapeutic areas (342.1 M€) and 10.2% from other non-remunerated pharmaceutical services with value for the society (89.5 M€). These other services comprise community pharmacists’ contributions to medicines wastage management, syringe-exchange program, voluntary participation in the *Valormed* program (medicines disposal program), research projects and contributions to pharmaceutical students’ curricular internships. Together, the services on medicines wastage and syringe-exchange programs are estimated to generate a value of 76.1 M€ to society.

Previous results can be further detailed in three major health areas: chronic conditions/therapies, mother and child health and transversal services, which may have held societal values of 187.6 M€, 13.3 M€ and 589.3 M€, respectively. Services provided in chronic conditions/therapies are estimated to have a positive impact of 8.0% increment in QoL, having allowed 120,604 additional QALYs in 2014. Moreover, these sets of services are estimated to avoid 1,480,442 physician visits, 13,101 emergency-room visits and 12,962 hospitalisations. Mother and child health services are estimated to cover annually 654,285 mothers or children and to provide a 0.2% increase in QoL, with an added economic value of 13.3 M€. Evidence suggests that mother and child services do not impact health resource utilisation and therefore the economic value provided derives exclusively from non-remunerated community pharmacies services. Transversal services include services in several areas, such as non-prescription medicines counselling, multidose drug dispensing or domiciliary support. These services are estimated to increase QoL in 12.7% and hold an economic value to society of 589.3 M€ (266.3 M€ in non-remunerated community pharmacies services and 323.0 M€ in avoided costs with health resource utilisation). In this specific setting (transversal services), a total of 4,510,665 physician visits, 8953 emergency-room visits and 9446 hospitalisations were estimated to have been avoided due to community pharmacies services.

### Services in specific chronic conditions

Within chronic conditions, the two areas where community pharmacies services were most valued were hypertension and diabetes. Services provided in hypertension are estimated to cover 828,216 patients and to be associated with an average reduction of 14.5 mmHg (standard error: 4.0 mmHg) in mean systolic blood pressure. A QoL increment of 5.7% is estimated in the covered population, representing a benefit of 33,129 additional QALYs. Other social benefits comprise 1,549,094 avoided physician visits, 7557 avoided emergency-rooms visits and 7557 avoided hospitalisations. Estimated added economic value totalizes 147.6 M€: 119.3 M€ representing savings in health resources’ utilisation and 28.3 M€ provided by non-remunerated community pharmacies services.

Regarding services in diabetes, a total of 286,186 covered patients were estimated with a decrease in glycated haemoglobin of 0.7 percentage points. The estimated social value achieved a 4.7% gain in QoL and 10,707 additional QALYs. The utilisation of pharmacy services was estimated to avoid 279,807 medical acts: 274,577 physician visits, 2615 emergency-room visits and 2615 hospitalisations.

### Potential future services

Pharmaceutical services potentially considered in the near future but not yet provided in community pharmacies, such as, repeat dispensing, patient call-back systems or medication reconciliation (from inpatient to outpatient setting) were also evaluated.

Overall future community pharmacies services may increase QoL by 6.9% in 1,724,274 additional patients to be potentially covered (16.6% of the Portuguese population). This increment could possibly be translated in 75,640 additional QALYs. Moreover, it could eventually have an impact on health resource consumption, avoiding 363,608 physician visits, 39 emergency-room visits and 39 hospitalisations. The added economic value of the broader range of potential future community pharmacies services may achieve 144.8 M€ (Table 2).

**Table 2:** Social and economic value of the future community pharmacies services

*Potential future services*			
	With service	Without service	Difference
Population Covered (*n*)	1,724,274	1,724,274	0
Estimated number of pharmaceutical services (*n*)	46,870,802	0	46,870,802
Time spent in services (*hours*)	3,807,870	0	3,807,870
Social Value			
Quality of Life			
Quality of Life (growth rate, %)	0.684	0.640	0.044 (+6.9%)
Quality adjusted life years	1,178,882	1,103,242	75,640
Health Resource Utilisation (n)	8,079,565	8,443,250	−363,685
Physician visits (n)	6,848,352	7,211,960	−363,608
Emergency-room visits (n)	1,215,835	1,215,873	−38
Hospitalisations (n)	15,379	15,417	−38
Economic Value			
Total cost (€)	458.1 M€	602.9 M€	−144.8 M€
Community pharmacy (€)			
Community pharmacist’ service (monetary compensated and non-monetary compensated)	−120.3 M€	0 €	−120.3 M€
Patients	0 €	0 €	0 €
Cost of Health Resource Utilisation (€)			
Physician visits	459.0 M€	483.3 M€	−24.4 M€
Emergency-room visits	87.4 M€	87.4 M€	0.0 M€
Hospitalisations	32.1 M€	32.2 M€	−0.1 M€

M€, Million euros

The evaluation of the potential future services can be further divided in three categories: integration with primary care, integration with secondary care and transversal services.

Integration with primary care could lead to an increase in QoL by 4.0% and a gain of 23,116 additional QALYs. The estimated economic value provided to society could sum up to 110.2 M€, which is the result of 102.5 M€ in non-remunerated pharmaceutical services, 7.7 M€, 2.8 thousand € and 81.721 thousand € in avoided physician visits, emergency-room visits and hospitalisations, respectively.

Integration with secondary care contemplated the possibility of community pharmacies to dispense medicines currently dispensed exclusively at hospital pharmacies or to adjust dosing regimens in anticoagulation therapy, among others. These sets of services are estimated to increase by 10.2% the QoL of the potentially covered population (835,703 patients) and to add 52,309 QALYs. The economic value provided to society could be the result of non-remunerated community pharmacies services (11.0 M€) and prevented physician visits (16.7 M€), summing 27.7 M€. Finally, transversal services might benefit the society in 6.8 M€ and lead to a 1.1% increase in 26,185 patients’ QoL.

### Discussion

From 2011 until 2014, Portugal was subject to an international financial assistance program which led to the implementation of several austerity measures impacting the socio-economic conjecture of the country [40]. The Portuguese National Health Service has recently celebrated 35 years of existence, health policy challenges to sustain the basic principles of its foundation – universal and general coverage, equity in access and tending towards free services – have been in the horizon [41]. In order to preserve such values, it is urgent to efficiently allocate resources and to promote structural reforms in health care services.

Community pharmacists already play a crucial role in Public Health and their widespread network of services is an important asset in access to healthcare. Therefore, the purpose of this study was to estimate the social and economic value of current and potential future community pharmacies services in health care, in Portugal.

From the social perspective, the current estimated annual value of pharmaceutical services in the community is an overall increment in QoL of 8.3%, representing 260,245 additional QALYs. Moreover, potential future services could be accountable for an additional increase of 6.9% in QoL and 75,650 QALYs.

The economic value of community pharmacies services was demonstrated by the generated 879.6 M€ savings, attributable mainly to the health resource utilisation averted and the non-remunerated services of pharmacists. Putting these results into context, the generated 879.6 M€ savings represented 5.6% of the Portuguese Public Health expenditure and 0.5% of the GDP, in 2014 [3]. Savings could amount to more 144.8 M€ if one considers the potential future integration of community pharmacies in primary and secondary care services and other transversal services.

This is the first study that has ever suggested social and economic value for nationwide services of community pharmacies. Previously published systematic reviews also demonstrated clinical, social and economic benefits of specific community pharmacies services worldwide [16, 18, 42–44]. Overall assessment produced a positive impact on clinical, social and economic endpoints, which is in line with the present study results. Most international studies, focused on particular diseases or conditions, showed favourable results in clinical endpoints such as glycated haemoglobin, lipid parameters or blood pressure [11–13, 21, 45], which was concordant with other Portuguese published studies [46–51]. These studies reported mainly outcomes in patients with diabetes, hypertension, dyslipidaemia, asthma or COPD. For example, a meta-analysis of pharmaceutical services showed a decrease in glycated haemoglobin of 1.8%, whereas this study conducted in Portugal suggest a reduction of 0.7%; and for systolic and diastolic blood pressure, this meta-analysis presented a mean decrease of 7.8 mmHg and 2.9 mmHg, respectively, which were higher than the reductions considered in this study of 3.4 mmHg and 1.5 mmHg, respectively [17, 48]. Services in weight management, smoking cessations or adherence by both national and international literature were reported in other studies and also evaluated in this economic model [49, 52, 53]. A recent systematic review of cost-effectiveness of professional pharmacy services concluded that community pharmacies services may improve patients’ health with a beneficial economic impact to health care systems, which was in line with the current study [44].

Throughout the literature review it was noteworthy the surplus of studies conducted in the United Stated of America (USA), in comparison with other countries. In fact, USA was pioneer in implementing a set of measures to acknowledge community pharmacies and their role in providing pharmaceutical care, such as the possibility for flu vaccine administration and other health care programmes [18, 54]. This set of measures promotes the integration of pharmacists in the health care team and advocates for the provision of pharmaceutical care [17]. Another study conducted in Ireland showed that favourable policy incentives may lead to a better quality of pharmaceutical services [55], reinforcing the contribution of each countries’ pharmaceutical policies in promoting services with great value to the health care system.

Being a pioneer research in this field, this study bears limitations and caveats that must be addressed. First of all, community pharmacies services have existed for several decades. Hence, a scenario which simulates the absence of pharmaceutical services is almost impossible to achieve, and so no natural comparator scenario was possible. On the other hand, this study also anticipated some of the consequences that hypothetical future services might have in health care.

Despite the assumptions associated with future services, it is important to point out that some of the included services had already been provided or piloted in Portugal and others are already delivered in other countries. In the latter case, simulations were supported by studies previously conducted in other countries and validated by the expert panel. As such, results presented must be interpreted having these limitations into consideration.

Throughout the conducted literature review, there was a limited availability of studies reporting QoL and health resource utilisation. Moreover, it was observed that the likelihood of reporting QoL and economic data was associated with the robustness of the service that was consequently associated with positive outcomes, which in itself may be a source of bias [11, 12, 21]. Despite this limitation, data retrieved from internationally performed services were always subjected to expert validation, in order to adjust data to the Portuguese context.

Published evidence of pharmaceutical care is mostly derived from USA [17, 18, 56], whose health care system presents major differences in comparison with Europe. Nevertheless, accounted differences regarding health resource consumption were adjusted to the Portuguese context by national expert physicians. Also, costs associated with health resource utilisation were exclusively retrieved from national sources.

Despite the limitations, the expert panel was granted the possibility to exclude services not considered relevant in the national setting. Consequently, the developed model included exclusively pertinent services provided by Portuguese community pharmacists, validated by Portuguese experts, which could reduce the potential impact that outcomes of services conducted internationally might have when transposed to the national context.

Furthermore, this study evaluated, simultaneously, a substantial amount of services from three major perspectives: clinical, social and economic. The definition of clinical outcomes allowed a micro evaluation of each service separately. Further aggregation and comparison of services was possible due to the inclusion of macro outcomes, such as QoL (social) and costs associated with health resource utilisation (economic).

Another accounted advantage was the contribution of CEFAR that provided real-world data on services conducted and prices charged to the patients, within a representative sample of community pharmacies (more than 75% of Portuguese community pharmacies). Although much of the research conducted by CEFAR was not published, it represents the national reality and may reduce the impact of publication bias.

Moreover, the cost of pharmaceutical activities and time spent with services used to estimate non-remunerated pharmaceutical services were retrieved from previously conducted studies in Portugal with a representative sample of community pharmacies, reducing the potential impact that services led elsewhere could have had on the developed model [33–35].

Further studies with sound clinical, social and economic outcomes are needed in order to build a growing body of evidence of cost-effectiveness of community pharmacies services. Accurate real-world studies, with control groups, evaluating clinical, social and/or economic outcomes of pharmaceutical services in several therapeutic areas, for instance, diabetes, hypertension, asthma or dyslipidaemia, should be promoted in order to update the current model with services conducted in the Portuguese real-life context and real-world data.

In 2007, the Portuguese legislation recognised community pharmacies as adequate sites to provide pharmaceutical services, allowing several services to be provided by community pharmacists [57, 58]. In 2014, an agreement between National Association of Pharmacies and the Ministry of Health established several services such as the restart of the syringe-exchange program in community pharmacies, and the promotion of medication adherence in diabetes patients to promote Public Health [59]. More recently, the government has established the intention to enhance community pharmacies services in order to promote the rational utilisation of medicines, close relationship with the National Health System and delegate services currently available at hospital level, namely the supply of medicines for oncology and transmissible diseases, currently exclusively available at hospital pharmacies [32].

## Conclusions

In conclusion, this study highlights the importance of community pharmacies services in health care, revealed by the high social impact on patients’ QoL and the substantial economic value to society. Similarly to other countries, policy and economic incentives to pharmaceutical services conducted in community pharmacies are justified and may further lead to an increased social and economic benefit on a broader range of patients.

## Additional files

Additional file 1: Table S1. Current community pharmacist’s services evaluated. (DOCX 26 kb)

Additional file 2: Table S2. Future community pharmacist’s services evaluated. (DOCX 24 kb)

Additional file 3: Table S3. Search strategy. (DOCX 28 kb)

## Abbreviations

CEFAR: Centre for Health Evaluation & Research
COPD: Chronic obstructive pulmonary disease
DRG: Diagnosis-related groups
GDP: Gross Domestic Product
HIV: Human immunodeficiency virus
M €: Million euros
QALYs: Quality-adjusted life years
QoL: Quality of life
USA: United States of America
