# Supplementary material for: Social and economic value of Portuguese community pharmacies in health care
Source: BMC Health Serv Res. 2017 Aug 29;17:606. doi: 10.1186/s12913-017-2525-4 (PMC5576248; doi:10.1186/s12913-017-2525-4)
Supplement: Supplementary file 1 — Current community pharmacist’s services evaluated. (DOCX 26 kb) [file 12913_2017_2525_MOESM1_ESM.docx]

## **S1 Table Current community pharmacist’s services evaluated**

| **Current Community Pharmacist’s Services in Health Care** | | |
| --- | --- | --- |
| **Chronic conditions/therapies** | **Asthma** | - Disease management - Teaching inhalation technique - Adherence promotion - Campaigns to identify non-controlled patients |
|  | **COPD** |  |
|  | **Diabetes** | - Disease management - Adherence promotion - Campaigns to identify non-controlled patients |
|  | **Dislipidemia** |  |
|  | **High Blood Pressure** |  |
|  | **Coagulation disorders** | - Disease management - Counselling/ disease or therapy education - Monitoring clinical parameters |
|  | **Obesity** | - Counselling/ disease or therapy education - Monitoring clinical parameters - Campaigns to identify obese patients |
| **Mother and child e health** | **Pregnancy/ Breastfeeding** | - Counselling - Teaching utilisation technique of care materials for pregnant women and their newborns - Pregnancy test |
|  | **Children** | - Counselling - Teaching utilisation technique of care materials for children |
| **Transversal interventions** | **Administration/**  **Provision** | - Medicines (including injectable) - First-aid - Vaccines (not included in National Vaccination Program) |
|  | **Domiciliary/nursing home support** | - Domiciliary support - Domiciliary drug delivery |
|  | **Counselling** | - Dermocosmetics/Medical devices - Non-prescription medicines - Dietary supplement - Medicines for veterinary use |
|  | **Sun protection** | - Counselling - Campaigns |
|  | **Programs** | - Syringe-exchange - Smoking cessation |
|  | **Academic** | - Investigation projects - Curricular internships |
|  | **Medicines-related** | - Multidose drug dispensing - Brown-bag revision (medication review) - Identification of prescription errors - Pharmacovigilance - Compounding - Medicines wastage management |
|  | - Medicines disposal program **(*Valormed program*)** | |
