# Supplementary material for: Social and economic value of Portuguese community pharmacies in health care
Source: BMC Health Serv Res. 2017 Aug 29;17:606. doi: 10.1186/s12913-017-2525-4 (PMC5576248; doi:10.1186/s12913-017-2525-4)
Supplement: Supplementary file 2 — Future community pharmacist’s services evaluated. (DOCX 24 kb) [file 12913_2017_2525_MOESM2_ESM.docx]

## **S2 Table Potential future community pharmacist’s services evaluated**

| **Potential Future Community Pharmacist’s Services in Health Care** | |
| --- | --- |
| **Integration with secondary care** | - **Dosing adjustments in anticoagulation therapy** |
|  | - **Community pharmacy dispensing of medicines currently dispensed exclusively at hospital pharmacy** |
|  | - **Disease management – arthritis** |
|  | - **Early detection of Human Immunodeficiency Virus (HIV)** |
|  | - **Medication Reconciliation** (transition between inpatient and outpatient setting) |
|  | - **Directly Observed Therapy (DOT) in tuberculosis** |
| **Integration primary care** | - **Travel counselling** |
|  | - **Pain medication management therapy** |
|  | - **Early osteoporosis detection** |
|  | - **Disease management – depression** |
|  | - **Disease management – rhinitis** |
|  | - **Repeat prescribing** |
| **Transversal interventions** | - **Teaching the correct technique in the utilisation of several devices (apps and other technologies)** |
|  | - **Patient call-back system** |
|  | - **Opioid replacement therapy programs** |
