# Supplementary material for: Social and economic value of Portuguese community pharmacies in health care
Source: BMC Health Serv Res. 2017 Aug 29;17:606. doi: 10.1186/s12913-017-2525-4 (PMC5576248; doi:10.1186/s12913-017-2525-4)
Supplement: Supplementary file 3 — Search strategy. (DOCX 28 kb) [file 12913_2017_2525_MOESM3_ESM.docx]

## **S3 Table Search strategy**

| # | PubMed search |
| --- | --- |
|  | “Pharmaceutical services” [mh] OR “Pharmaceutical service*” [tiab] OR “pharmacy service*” [tiab] OR “pharmaceutic service*” [tiab] OR “pharmaceutical care*” [tiab] OR “pharmacy care*” [tiab] OR “pharmaceutic care*” [tiab] OR “pharmacy intervention*” [tiab] OR “pharmaceutical intervention*” [tiab] OR “pharmaceutic intervention*” [tiab] OR services/interventions [tiab] OR interventions/services [tiab] OR “pharmacy practice” [tiab] OR Pharmacies [mh] OR “community pharmacy” [tiab] OR “community pharmacies” [tiab] OR pharmacy-based [tiab] OR “independent pharmacy” [tiab] OR “independent pharmacies” [tiab] OR “retail pharmacy” [tiab] OR “retail pharmacies” [tiab] OR “chain pharmacy” [tiab] OR “chain pharmacies” [tiab] OR “clinical pharmacy” [tiab] OR “ambulatory pharmacy” [tiab] OR “ambulatory pharmacies” [tiab] OR “ambulatory setting” [tiab] OR Pharmacists [mh] OR “community pharmacist*” [tiab] OR “pharmacist* intervention” [tiab] OR “pharmacy practitioner*” [tiab] OR “pharmacist care” [tiab] |
|  | Asthma [mh] OR Asthma [tiab] OR “Pulmonary Disease, Chronic Obstructive” [mh] OR “Chronic obstructive pulmonary disease” [tiab] OR COPD [tiab]) AND (management [tiab] OR control [tiab] OR education [tiab] OR care [tiab] OR plan [tiab] OR intervention [tiab] OR service [tiab])) OR “inhaler technique” [tiab] OR “inhalation technique” [tiab] OR Rhinitis [mh] OR Rhinitis [tiab] OR Rhinitides [tiab]) AND (management [tiab] OR control [tiab] OR education [tiab] OR care [tiab] OR plan [tiab] OR intervention [tiab] OR service [tiab] OR “tobacco use cessation” [mh] OR “tobacco use cessation” [tiab] OR “Smoking Cessation” [tiab] OR “Smoking-Cessation” [tiab] OR “giving up smoking” [tiab] OR “quit* smoking” [tiab] OR “tobacco cessation” [tiab] OR “nicotine therapy” [tiab] OR “nicotine replacement” [tiab] OR “lipid management” [tiab] OR “cholesterol risk” [tiab] OR “lipid screening” [tiab] OR “lipid profile” [tiab] OR “cholesterol test*” [tiab] OR “cholesterol screening” [tiab] OR “cholesterol measurement” [tiab] OR “Cholesterol level*” [tiab] OR “total blood cholesterol” [tiab] OR "cardiovascular risk" [tiab] OR “Cardiovascular disease management” [tiab] OR “cholesterol risk management” [tiab] OR “cholesterol management” [tiab] OR “triglyceride* management” [tiab] OR “triglyceride* screening” [tiab] OR “triglyceride* profile” [tiab] OR “triglyceride* test*” [tiab] OR “triglyceride* measurement” [tiab] OR “triglyceride* level*” [tiab] OR “glycemic control” [tiab] OR “glycaemic control” [tiab] OR “diabetes control“ [tiab] OR “glucose control” [tiab] OR “diabetes management” [tiab] OR “diabetes follow-up” [tiab] OR “monitoring of blood glucose” [tiab] OR “diabetes monitoring” [tiab] OR “diabetes education” [tiab] OR “diabetes care” [tiab] OR “diabetes service*” [tiab] OR “Insulin Pens” [tiab] OR “insulin device*” [tiab] OR “insulin medical device*” [tiab] OR “insulin administration*” [tiab] OR “hypertension management” [tiab] OR “hypertension control” OR “hypertension care” [tiab] OR “hypertension screening” [tiab] OR “hypertension follow-up” [tiab] OR “blood pressure” [tiab] OR “Blood Coagulation Tests” [mh] OR “Blood Coagulation Tests” [tiab] OR “INR monitoring” [tiab] OR “INR control” OR “warfarin education” [tiab] OR “warfarin management” [tiab] OR “warfarin control” [tiab] OR “warfarin treatment” [tiab] OR “warfarin service*” [tiab] OR “anticoagulation management” [tiab] OR “anti-coagulation management” [tiab] OR “anticoagulation education” [tiab] OR “anti-coagulation education” [tiab] OR “weight management” [tiab] OR weight-management [tiab] OR “obesity control” [tiab] OR “weight loss” [tiab] OR “weight control” [tiab] OR “counterweight pharmacy program*” [tiab] OR “counterweight program*” [tiab] OR ((“Abdominal perimeter” [tiab] OR “body mass index” [tiab] OR BMI [tiab]) AND (measurement* [tiab] OR assess* [tiab] or management* [tiab] OR program* [tiab] OR service [tiab])) OR “Medicine waste” [tiab] OR “Returned medicine” [tiab] OR OR “Return of unwanted medicines” [tiab] OR “drug take-back program*” [tiab] OR “drug take-back service” [tiab] OR “medication-take back” [tiab] OR Vaccination [mh] OR Vaccination [tiab] OR immunization [tiab] OR Vaccines [mh] OR vaccines [tiab] OR “parenteral drugs” [tiab] OR “Injectable administration” [tiab] OR “parenteral administration” [tiab] OR “Needle-Exchange programs” [mh] OR “Needle-Exchange program*” [tiab] “Needle Exchange program*” [tiab] OR NEP [tiab] OR “needle-exchange service” [tiab] OR “needle exchange service” [tiab] OR “Syringe exchange program*” [tiab] OR “Syringe-exchange program*” [tiab] OR SEP [tiab] OR “needle-syringe program*” [tiab] OR “Needle and Syringe Program*” [tiab] OR NSP [tiab] OR “Sunscreening Agents” [mh] OR “Sunscreen*” [tiab] OR “solar protection” [tiab] OR “sun protection” [tiab] OR “ ultraviolet radiation” [tiab] OR “skin cancer” [tiab] OR melanoma [tiab]) AND (prevention [tiab] OR program* [tiab] OR screening [tiab] OR intervention [tiab] OR campaign [tiab] OR awareness [tiab] OR “nutrition* consult*” [tiab] OR “nutrition* advice*” [tiab] OR “nutrition* assessment*” [tiab] OR “podiatry” [tiab] OR “physiotherapy” [tiab] OR “diabetic foot” [tiab] OR “Pregnancy Tests” [mh] OR “pregnancy test*” [tiab] OR Hyperuricemia [mh] OR Hyperuricemia [tiab] OR “uric acid” [tiab] OR gout [tiab]) AND (management [tiab] OR risk [tiab] OR screening [tiab] OR measurement [tiab] OR level* [tiab] OR test* [tiab] OR “early detection” [tiab] OR education [tiab] OR Hemoglobins [mh] OR Hemoglobin* [tiab] OR Haemoglobin* [tiab] OR anemia [tiab] OR anaemia [tiab]) AND (management [tiab] OR risk [tiab] OR screening [tiab] OR measurement [tiab] OR level* [tiab] OR test* [tiab] OR “early detection” [tiab] OR education [tiab] OR “Prostate-Specific Antigen” [mh] OR “Prostate-Specific Antigen” [tiab] OR PSA [tiab] OR “prostate cancer” [tiab]) AND (risk [tiab] OR screening [tiab] OR measurement [tiab] OR level* [tiab] OR test* [tiab] OR “early detection” [tiab] OR education [tiab]) OR “Alanine Transaminase” [mh] OR “Alanine Transaminase” [tiab] OR “glutamic-pyruvate transaminase” [tiab] OR GPT [tiab]) AND (management [tiab] OR screening [tiab] OR measurement [tiab] OR level* [tiab] OR test* [tiab] OR “early detection” [tiab] OR education [tiab] creatinine [tiab] OR “renal function” [tiab] OR “renal impairment” [tiab] OR “chronic kidney disease” [tiab] OR CKD [tiab]) AND (management [tiab] OR risk [tiab] OR screening [tiab] OR measurement [tiab] OR level* [tiab] OR test* [tiab] OR “early detection” [tiab] OR education [tiab] hearing [tiab] OR audio* [tiab]) AND (screening [tiab] OR evaluaton* [tiab] OR test* [tiab] OR loss [tiab] OR “early detection” [tiab] OR “biologic component*” [tiab] OR “biologic specimen*” [tiab] OR “biological component*” [tiab] OR “biological specimen*” [tiab] OR blood [tiab] OR urine [tiab] OR faeces [tiab] OR saliva [tiab]) AND collect* [tiab] OR “Drugs, Generic” [mh] OR “generic drug*” [tiab] OR “minor ailment*” [tiab] OR PMAS [tiab] OR MAS [tiab] OR “common illness*” [tiab] OR “self-limiting illness*” [tiab] OR “common condition*” [tiab] OR “self-limiting condition*” [tiab] OR “over-the-counter” [tiab] OR “over the counter” [tiab] OR OTC [tiab]) AND (scheme* [tiab] OR service* [tiab] OR auto-administration [tiab] OR self-administration [tiab] OR “self administration” [tiab] OR auto-injectable [tiab] OR auto-injector* [tiab]) AND (training* [tiab] OR education* [tiab] OR program* [tiab] OR intervention [tiab] OR campaign [tiab] OR self-management [tiab] OR self-surveillance [tiab] OR auto-management [tiab] OR auto-surveillance [tiab]) AND (training* [tiab] OR education* [tiab] OR program* [tiab] OR intervention [tiab] OR campaign [tiab] OR “Medication Therapy Management” [mh] OR “Therapy Management” [tiab] OR “Medication Management” [tiab] OR “Medication review*” [tiab] OR “therapeutic drug monitoring” [tiab] OR “patient medication records” [tiab] OR “medication counselling” [tiab] OR “treatment monitoring” [tiab] OR “Home Medicines Reviews” [tiab] OR “target drug program” [tiab] OR “Drug dosing service” [tiab] OR “dosing adjustment service” [tiab] OR polymedicat* [tiab] OR “drug administration” [tiab] OR “administration of drug*” [tiab] OR “disease management” [tiab] OR “case management” [tiab] OR “patient monitoring” [tiab] OR “screening services” [tiab] OR “patient follow-up” OR “disease follow-up” OR “patient group direction” [tiab] OR “Drug-Related Side Effects and Adverse Reactions” [mh] OR “Drug-Related Problem*” [tiab] OR “medication-related problems” [tiab] OR” drug safety” [tiab] OR “dosing inadequacy” [tiab] OR “drug dosing error” [tiab] OR “adverse drug reaction” [tiab] OR ADR [tiab] OR “adverse reaction” [tiab] OR “adverse event*” [tiab] OR “adverse drug event*” [tiab] OR “drug toxicit*” [tiab] OR “drug side effect*” [tiab] OR “medication error*” [tiab] OR “prescribing error*” [tiab] OR “prescription error*” [tiab] OR “brown bag medication review*” [tiab] OR “brown-bag medication review*” [tiab] OR “brown bag review*” [tiab] OR “brown-bag review*” [tiab] OR “Brown bag counseling” [tiab] OR “Brown-bag counseling” [tiab] OR “brown bag check-up” [tiab] OR “brown-bag check-up” [tiab] OR “brown bag program*” [tiab] OR “brown-bag program*” [tiab] OR “drug utilization review” [tiab] OR “medication review*” [tiab] OR “Drug-dispensing system*” [tiab] OR “Drug-dispensing service*” [tiab] OR “Drug dispensing system*” [tiab] OR “Drug dispensing service*” [tiab] OR “Dose-dispensing system*” [tiab] OR “Dose-dispensing service*” [tiab] OR “Dose dispensing system*” [tiab] OR “Dose dispensing service*” [tiab] OR “home care” [tiab] OR “home support” [tiab] OR “home service*” [tiab] OR “nursing home care” [tiab] OR “nursing home support” [tiab] OR “nursing home service*” [tiab] OR “residence care” [tiab] OR “residence support” [tiab] OR “residence service*” [tiab] OR “Medication Reconciliation” [mh] OR “Medication Reconciliation*” [tiab] OR “transitions of care” [tiab] OR “transition to ambulatory” [tiab] OR “ambulatory care transition” [tiab] OR “care transition*” [tiab] OR “pharmacy-only refill program” [tiab] OR “health services” [mh] OR “health services” [tiab] OR “patient care” [mh] OR “patient care service*” [tiab] OR “clinical care service” [tiab] OR “Health Education” [mh] OR “health education” [tiab] OR “health promotion” [tiab] OR “promotion of health” [tiab] OR “disease education” [tiab] OR “therapy education” [tiab] OR “therapeutic education” [tiab] OR “Medication Adherence” [mh] OR Adheren* [tiab] OR Nonadheren* [tiab] OR Non-adheren* [tiab] OR “Non adheren*” [tiab] OR persisten* [tiab] OR “drug complian*” [tiab] OR drug-complian* [tiab] OR “medication complian*” [tiab] OR medication-complian* [tiab] OR “complian* to medication” [tiab] OR non-complian* [tiab] OR “non complian*” [tiab] OR noncomplian* [tiab] OR “Drug Compounding” [mh] OR “compounding” [tiab]OR “Drug Preparation*” [tiab] OR“Veterinary Drugs” [mh] OR “veterinary medicinal products” [tiab] OR “veterinary medicine*” [tiab] OR “veterinary drug*” [tiab] OR “ veterinary advice” [tiab] or “veterinary consultancy” [tiab] OR “internship*” [tiab] OR “academic education” [tiab] OR “pharmacy residency training” [tiab] OR “fellowship*” [tiab] OR “student training” [tiab] OR traineeship [tiab] OR “pharmacy trainee” [tiab] OR “pharmaceutic trainee” [tiab] OR research [tiab] OR “non-interventional studies” [tiab] OR “non interventional studies” [tiab] OR “observational studies” [tiab] OR “regulatory studies” [tiab] OR “first-aid kits” [tiab] OR “first aid kits” [tiab] “first aid” [mh] OR “first aid*” [tiab]) AND (administration [tiab] OR service [tiab] OR practice [tiab])) OR (“wound management” [tiab] OR “wound care” [tiab] OR “wound dressing” [tiab] OR “burn management” [tiab] OR “burn care” [tiab] OR “burn dressing” [tiab]) OR “Drug Recalls” [mh] OR “drug recall” [tiab] OR drug-recall [tiab] OR "medication recall" [tiab] OR |
|  | “Osteoporosis Screening” [tiab] OR “Bone Mineral Density screening” [tiab] OR “Arthritis Management” [tiab] OR “bone mass measurement” [tiab] OR “bone density measurement” [tiab] OR “bone density evaluation” [tiab] OR “Bone density test*” [tiab] OR “methadone substitution program*” [tiab] OR “Opioid Dependence Treatment” [tiab] OR “Methadone maintenance treatment” [tiab] OR “HIV test*” [tiab] OR “HIV screen*” [tiab] OR “human immunodeficiency virus test*” [tiab] OR “human immunodeficiency virus screen*” [tiab] OR “Anti-Retroviral Agents” [mh] OR “Anti-Retroviral Agents” [tiab] OR “Anti Retroviral Agents” [tiab] OR “Antiretroviral Agents” [tiab] OR “ART” [tiab] OR “Anti-Retroviral Therapy” [tiab] OR “Anti Retroviral Therapy” [tiab] OR “Antiretroviral Therapy” [tiab] OR “cART” [tiab] OR “HAART” [tiab] OR “Antineoplastic Agents” [mh] OR “anticancer agents” [tiab] OR “Hepatitis C” [mh] OR “Hepatitis C” [tiab] OR “HCV” [tiab]) AND (dispens* [tiab] OR suppl* [tiab] OR access* [tiab] OR obtain* [tiab] OR provid* [tiab] OR receiv* [tiab] OR “alcohol cessation” [tiab] OR “alcohol interventions” [tiab] OR “Alcohol Screening” [tiab] OR “alcohol use disorder” [tiab] OR “Pain management” [mh] OR “Pain management*” [tiab] OR **“**Palliative Care” [mh] OR **“**Palliative Care” [tiab] OR “Palliative Treatment*” [tiab] OR “Palliative Therapy” [tiab] OR “palliative pharmaceutical care” [tiab] OR “Anti-Infective Agents, Urinary” [mh] OR “Urinary Antiseptics” [tiab] OR “self-management” [tiab] OR “self care” [mh] OR “self care” [tiab] OR Self-Care [tiab] OR Self-Management [tiab] OR “Self Management” [tiab] AND “health technology” [tiab] OR “biomedical technology” [mh] OR “biomedical technology” [tiab] OR “Biomedical Technologies” [tiab] OR “Health Care Technology” [tiab] OR “medical technology” [tiab] OR Mobile-health [tiab] OR “m-health” [tiab] OR “mhealth” [tiab] OR “mobile phone applications” [tiab] OR “Internet-based applications” [tiab] OR “E-health” [tiab] OR online [tiab] OR smartphones [tiab] OR “phone based” [tiab] OR phone-based [tiab] OR “mobile phone” [tiab]) OR “Repeat dispensing” [tiab] OR “repeat prescribing” [tiab] OR “medication refill” [tiab] OR medication-refill [tiab] OR callback [tiab] OR “Directly Observed Therapy” [mh] OR “Directly Observed Therapy” [tiab] OR DOT [tiab] OR “Directly observed treatment” [tiab] OR “travel health” [tiab] OR “travel information” [tiab] OR “pretravel health” [tiab] OR travel-health [tiab] OR “travel advice” [tiab] OR “travel vaccination*” [tiab] OR (renting [tiab] OR managing [tiab] OR rental [tiab]) AND (“medical devices” [tiab] OR “patient equipment” [tiab] OR breastfeeding [tiab] OR “breast pump*” [tiab] OR nebulizers [tiab]) OR “Diagnostic Techniques and Procedures” [mh] OR “Diagnostic Techniques and Procedures” [tiab] OR "diagnostic procedures" [tiab] OR "diagnostic test" [tiab] OR "therapeutic procedures" [tiab] |
|  | “quality of life” [mh] OR “quality of life” [tiab] OR “quality-of-life” [tiab] OR “QoL” [tiab] OR QL [tiab] OR “Life Qualities” [tiab] OR “Life Quality” [tiab] OR “patient reported outcomes” [tiab] OR “patient-reported outcomes” [tiab] OR PRO [tiab] |
|  | “Health Care Costs” [mh] OR cost* [tiab] OR econom* [tiab] |
|  | #2 OR #3 OR #4 OR #5 |
|  | #1 AND #6 |
|  | Filters: Humans, English, Portuguese |
